# Supplementary material for: Self-folding origami at any energy scale
Source: Nat Commun. 2017 May 18;8:15477. doi: 10.1038/ncomms15477 (PMC5454341; doi:10.1038/ncomms15477)
Supplement: Supplementary Information — Supplementary Figures, Supplementary Notes, Supplementary Methods and Supplementary References [file ncomms15477-s1.pdf]

Supplementary Figure 1

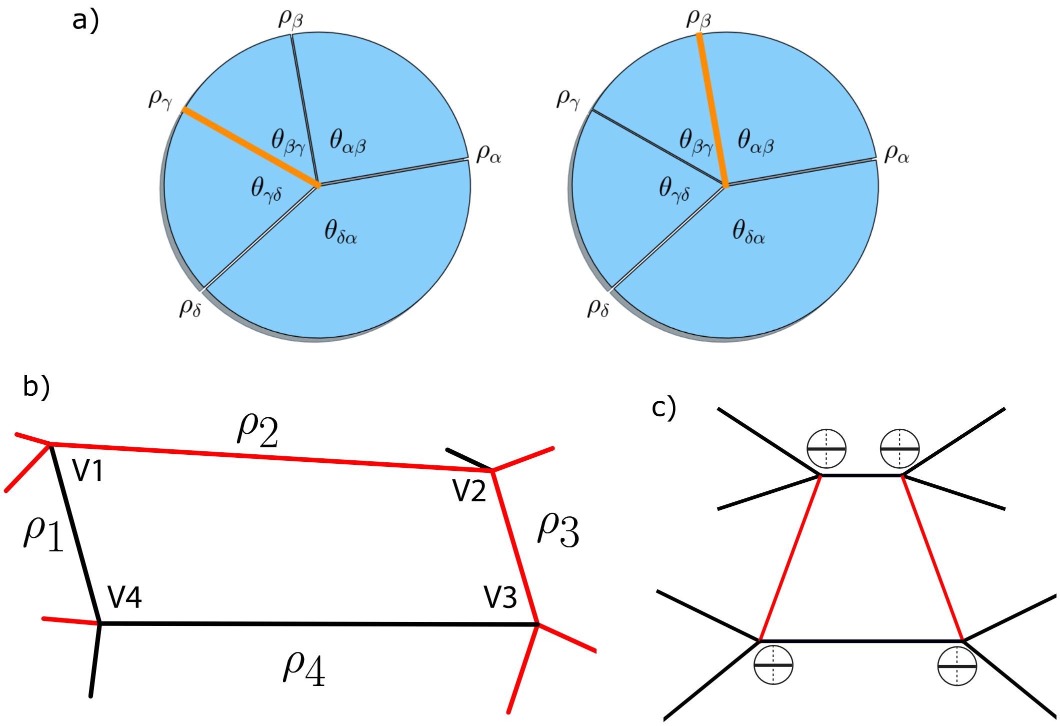

Supplementary Figure 1. **Modes of 4-vertices and quads.** (a) A 4-Vertex is the intersection of four creases labeled $\alpha, \beta, \gamma, \delta$. The in-plane angles between creases labeled with θ. The 4-vertex has to zero-energy folding modes, both of which have an 'odd-one-out' crease whose MV state is opposite to the rest. The odd-one-out crease must be picked such that the two neighboring in-plane angles sum to less than π (orange creases). (b) A quad is compose of four 4-vertices connected in a loop. Putative folding modes are uniquely defined by noting the MV choices for each crease (black - mountain, red - valley). (c) A trapezoidal quad that is rigid-foldable due to symmetry.

Supplementary Figure 2

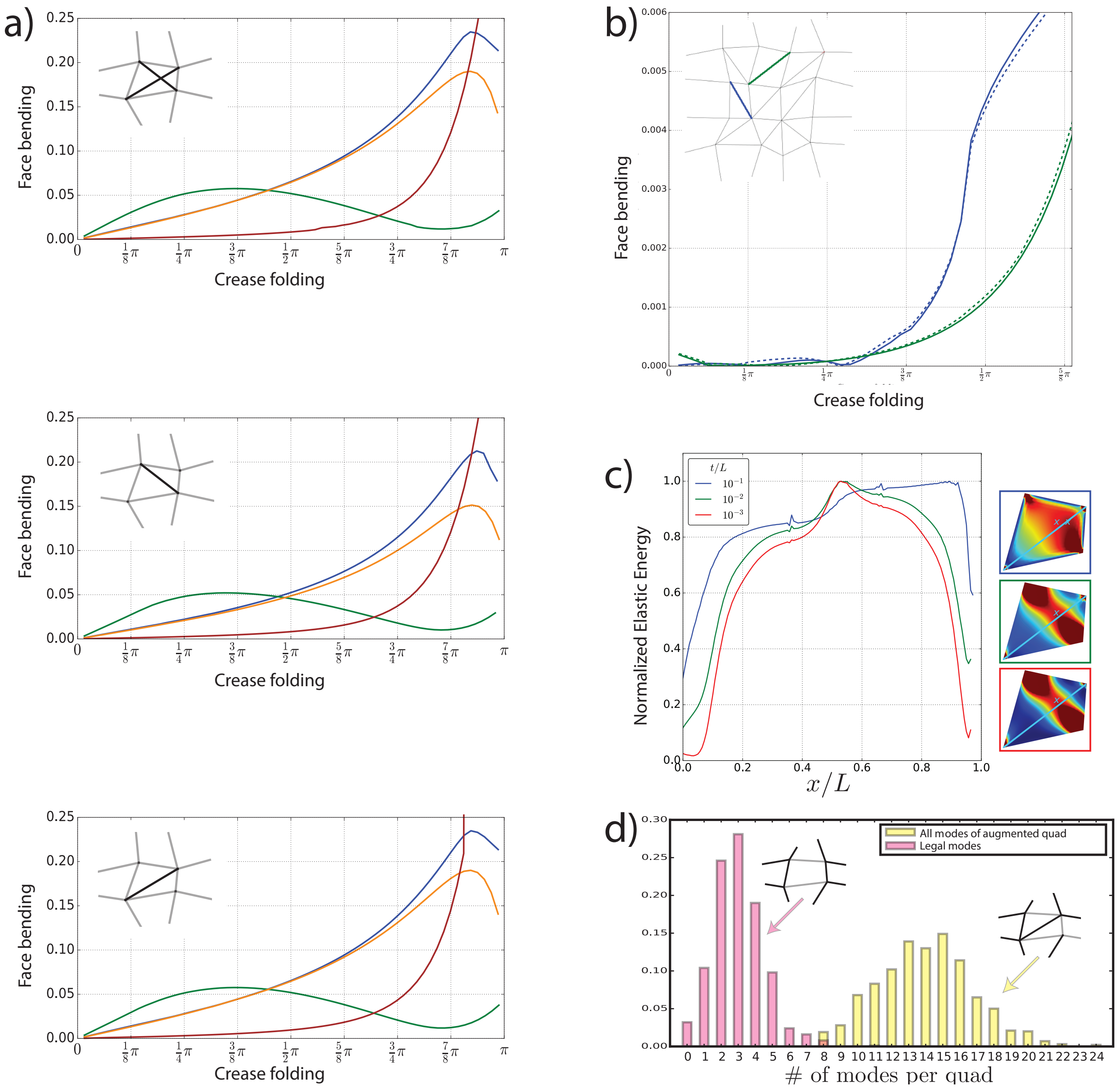

Supplementary Figure 2. **Folding of an added face diagonal is a robust proxy for face bending.** (a) We augment the quad studied in Fig. 5 of the paper with different face diagonals, seen in each inset. Whether augmented with the usual face diagonal, the other face diagonal or an X-shape double diagonal, face folding along different modes reported in Fig. 5a of the paper remains qualitatively the same (For the X-shaped double diagonal, we plot the average of two face folding angles). Any augmentation that adds one degree of freedom to the simple quad does not change its folding modes. (b) Face diagonals (and thus loop equations) applied to individual quads are useful in designing large origami patterns. Face folding of quads that are part of a large pattern (solid lines) closely approximates the behavior of each quad when 'cut out' and folded in isolation (dashed lines). Loop equations, applied quad by quad to tune foldability, can be used to design large patterns of desired foldability. (c) Finite element simulations of the center plate show that face diagonals are good approximations for thin origami patterns. Applying boundary conditions of a folding mode, the elastic energy is increasingly localized to a diagonal furrow as the thickness t of the plate is decreased relative to lateral dimensions L (Young's modulus $Y = 3\ GPa$ (material = PVC), $L \sim 10cm$. Simulations using COMSOL). (d) Augmented quads have multiple folding modes with distinct Mountain-Valley types. We sampled 1000 augmented quads and made a histogram of total number of folding motions (yellow bars); typical augmented quads have ~14 distinct modes. However, many of these, when restricted to the simple quad, are not legal folding motions (e.g., in right inset, one vertex has 2 valley and 2 mountain folds). We only consider modes of the augmented quad that induce legal MV patterns on the simple quad; the histogram (pink bars) indicates ~3 such modes for typical augmented quads. Diagonal face creases have been used before to study deformations of Miura lattices[1] and bistability of flat foldable crease patterns[2].

Supplementary Figure 3

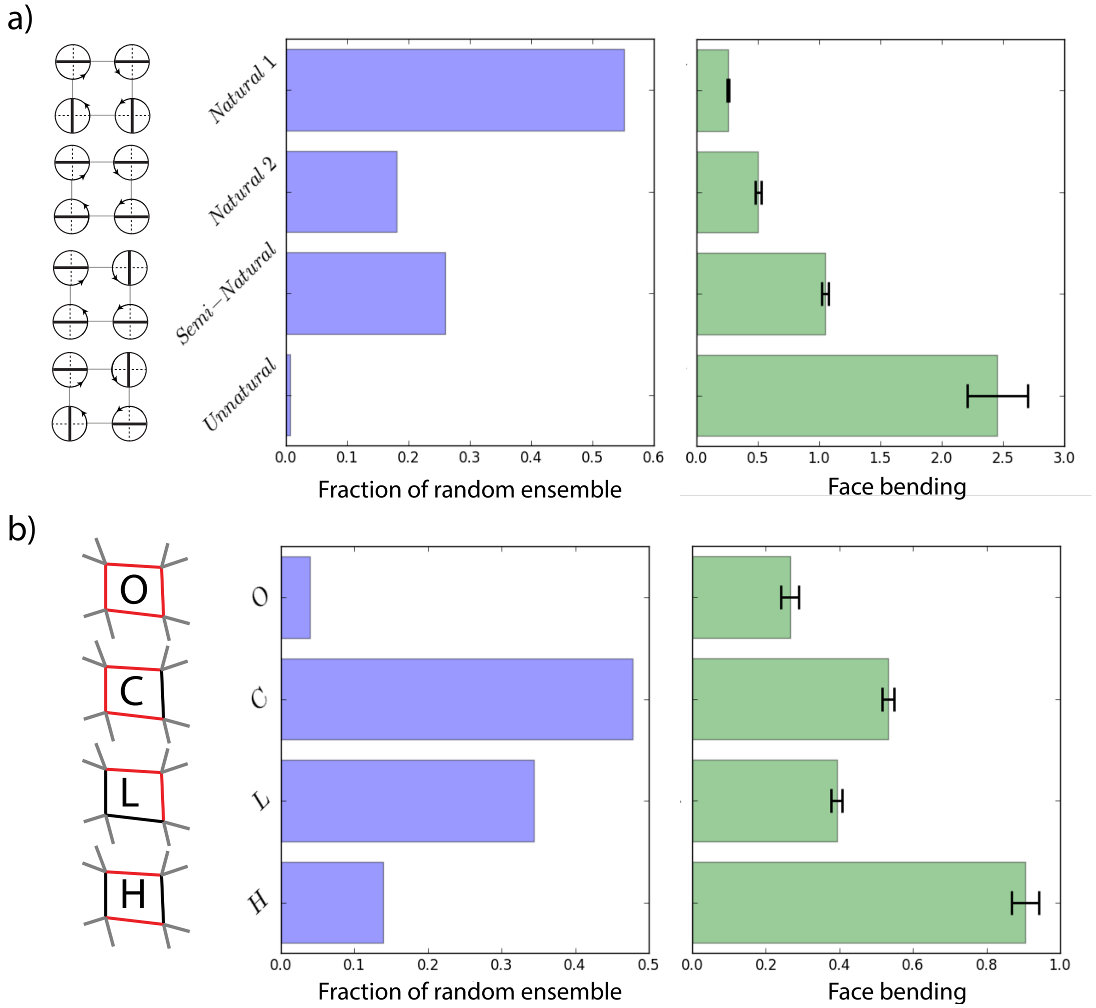

Supplementary Figure 3. **Classes of Mountain-Valley choices occur with widely varying frequency and face bending.** We generated 1000 random quads by displacing vertices randomly and folded them using random torques. (a) Most of the ensemble folded in the Natural 1 (~ 55%) or Semi-natural type (~ 28%) of Mountain-Valley type (blue bars). The average face bending (i.e., stiffness) during folding (green bars) was significantly higher for Unnatural and Semi-natural Mountain-Valley types, as also shown in Fig. 3 of the main paper. (b) We classified the same set of random quads by the type of Mountain or Valley data of the four internal creases. For example, quads of type O have internal creases of the same type - the MV states of external legs are ignored in this classification. C and L type quads are the most common (blue bars) and that H and C type quads are less foldable than others (green bars). Note that the two kinds of classification shown here in (a) and (b) are independent; e.g., a quad of type, say O, could be Natural,Semi-Natural or Unnatural and vice-versa.

Supplementary Note 1 - Vertex transfer and Loop equations

The basic unit of Origami consists of a quadrivalent vertex, the intersection of four creases. Creases are labeled with Greek letters α,β,γ,δ, where the angles between them (design space) are noted with θ, subscripted by the corresponding adjacent two creases. The angle to which a certain crease folds is given by ρ, subscripted with the corresponding crease label (see Supplementary Fig. 1a).

Any folded configuration of the vertex is given by the four crease folding (or dihedral) angles $\rho_\alpha, \rho_\beta, \rho_\gamma, \rho_\delta$. If we are given any one folding angle, say ρ_α, and the in-plane angles $\theta_{\alpha\beta}, \theta_{\beta\gamma}, \theta_{\gamma\delta}, \theta_{\delta\alpha}$, we can use spherical trigonometry to compute the remaining three fold angles $\rho_\beta, \rho_\gamma, \rho_\delta$.

In the main text, we wrote these equations symbolically in terms of transfer functions $T_{\alpha\beta}$. Here we present implicit formulas for these transfer functions in terms of $\mathrm{C}_\alpha \equiv \cos\rho_\alpha$:

Adjacent transfer relation $\alpha \to \beta$

$$\rho_\beta = T_{\alpha\beta}(\rho_\alpha; \{\theta\}):$$

$$(\mathrm{c}_{\beta\gamma}\mathrm{c}_{\gamma\delta} - \mathrm{s}_{\delta\alpha}\mathrm{s}_{\alpha\beta}\mathrm{C}_\alpha)^2 + (\mathrm{c}_{\gamma\delta}\mathrm{c}_{\delta\alpha} - \mathrm{s}_{\alpha\beta}\mathrm{s}_{\beta\gamma}\mathrm{C}_\beta)^2 - (\mathrm{c}^2_{\delta\alpha}+\mathrm{c}^2_{\beta\gamma}-1)(\mathrm{c}^2_{\alpha\beta}+\mathrm{c}^2_{\gamma\delta}-1)$$
$$= ((\mathrm{c}_{\delta\alpha}\mathrm{c}_{\alpha\beta} + \mathrm{s}_{\delta\alpha}\mathrm{s}_{\alpha\beta}\mathrm{C}_\alpha)(\mathrm{c}_{\alpha\beta}\mathrm{c}_{\beta\gamma} + \mathrm{s}_{\alpha\beta}\mathrm{s}_{\beta\gamma}\mathrm{C}_\beta) - (\mathrm{c}_{\delta\alpha}\mathrm{c}_{\beta\gamma} + \mathrm{c}_{\alpha\beta}\mathrm{c}_{\gamma\delta}))^2 \quad (1)$$

Transverse transfer $\alpha \to \gamma$

$$\rho_\gamma = T_{\alpha\gamma}(\rho_\alpha; \{\theta\}):$$

$$(\mathrm{c}_{\delta\alpha}\mathrm{c}_{\alpha\beta} + \mathrm{s}_{\delta\alpha}\mathrm{s}_{\alpha\beta}\mathrm{C}_\alpha) = (\mathrm{c}_{\beta\gamma}\mathrm{c}_{\gamma\delta} + \mathrm{s}_{\beta\gamma}\mathrm{s}_{\gamma\delta}\mathrm{C}_\gamma) \quad (2)$$

Reverse adjacent transfer $\alpha \to \delta$

$$\rho_\delta = T_{\alpha\delta}(\rho_\alpha; \{\theta\}):$$

$$(\mathrm{c}_{\alpha\beta}\mathrm{c}_{\beta\gamma} - \mathrm{s}_{\gamma\delta}\mathrm{s}_{\delta\alpha}\mathrm{C}_\delta)^2 + (\mathrm{c}_{\beta\gamma}\mathrm{c}_{\gamma\delta} - \mathrm{s}_{\delta\alpha}\mathrm{s}_{\alpha\beta}\mathrm{C}_\alpha)^2 - (\mathrm{c}^2_{\gamma\delta}+\mathrm{c}^2_{\alpha\beta}-1)(\mathrm{c}^2_{\delta\alpha}+\mathrm{c}^2_{\beta\gamma}-1)$$
$$= ((\mathrm{c}_{\gamma\delta}\mathrm{c}_{\delta\alpha} + \mathrm{s}_{\gamma\delta}\mathrm{s}_{\delta\alpha}\mathrm{C}_\delta)(\mathrm{c}_{\delta\alpha}\mathrm{c}_{\alpha\beta} + \mathrm{s}_{\delta\alpha}\mathrm{s}_{\alpha\beta}\mathrm{C}_\alpha) - (\mathrm{c}_{\gamma\delta}\mathrm{c}_{\alpha\beta} + \mathrm{c}_{\delta\alpha}\mathrm{c}_{\beta\gamma}))^2 \quad (3)$$

where $\mathrm{s}_{\alpha\beta} \equiv \sin(\theta_{\alpha\beta}), \mathrm{c}_{\alpha\beta} \equiv \cos(\theta_{\alpha\beta}), \mathrm{C}_\alpha \equiv \cos(\rho_\alpha)$ and similarly for all other factors. Note that, in these equations, we have adopted a convention in which at the flat unfolded state $\rho = \pi$ and hence $\mathrm{C} = -1$.

Linearization and branches: We will primarily use the more complex transverse transfer function $T_{\alpha\beta}$ between two consecutive creases. The transfer function can be linearized about the flat state, $\rho_\alpha = \pi - \epsilon\rho_\alpha$, to find

$$\epsilon\rho_\beta = R_{\alpha\beta}\,\epsilon\rho_\alpha, \text{ with } \boxed{R^2_{\alpha\beta} = \frac{-B \pm \sqrt{B^2 - 4AC}}{2A},} \quad (4)$$

with

$$A \equiv (\mathrm{s}_{\alpha\beta}\mathrm{s}_{\beta\gamma})^2(1 - (\mathrm{c}_{\delta\alpha}\mathrm{c}_{\alpha\beta} - \mathrm{s}_{\delta\alpha}\mathrm{s}_{\alpha\beta})^2) \quad (5)$$
$$B \equiv 2(\mathrm{s}_{\delta\alpha}\mathrm{s}_{\alpha\beta})(\mathrm{s}_{\alpha\beta}\mathrm{s}_{\beta\gamma})((\mathrm{c}_{\delta\alpha}\mathrm{c}_{\beta\gamma} + \mathrm{c}_{\alpha\beta}\mathrm{c}_{\gamma\delta}) - 2(\mathrm{c}_{\delta\alpha}\mathrm{c}_{\alpha\beta} - \mathrm{s}_{\delta\alpha}\mathrm{s}_{\alpha\beta})(\mathrm{c}_{\alpha\beta}\mathrm{c}_{\beta\gamma} - \mathrm{s}_{\alpha\beta}\mathrm{s}_{\beta\gamma})) \quad (6)$$
$$C \equiv (\mathrm{s}_{\delta\alpha}\mathrm{s}_{\alpha\beta})^2(1 - (\mathrm{c}_{\alpha\beta}\mathrm{c}_{\beta\gamma} - \mathrm{s}_{\alpha\beta}\mathrm{s}_{\beta\gamma})^2) \quad (7)$$

Note that we have two distinct solutions since Supplementary Equation (1) is quadratic in $\cos\rho_\alpha, \cos\rho_\beta$. This choice arises because a single vertex has two distinct folding modes[3]. We pick between these two branches by setting one of the two creases whose sum of angles around it are less than π (see Supplementary Fig. 1a), to be the odd-one-out in the Mountain/Valley pattern. For the vertex shown here, the two options for the odd-one-out crease are highlighted in orange. The larger value of R should be used when ρ_α and ρ_γ have opposite signs (i.e., are of opposite Mountain-Valley state). One intuitive explanation is that if the angles at the vertex were all $\frac{\pi}{2}$, R would be infinite if ρ_α, ρ_γ have opposite signs. The choice of branch for each vertex around the quad sets the MV class of the quad, as discussed in the main text.

Loop equation around a quadrilateral

Strategy

Now consider a quadrilateral made of four general vertices (Supplementary Fig. 1b). At each vertex a, we write the transfer equation between creases forming the sides of the quadrilateral, $\rho_i = T_a(\rho_{i+1})$.

We may rewrite this equation as $\mathbf{z}_a \equiv \rho_i - T_a(\rho_{i+1}) = 0$. As the quantity $\mathbf{z}_a$ vanishes for each vertex of the quadrilateral, we define $\mathbf{z} \equiv \sum_a \mathbf{z}_a = 0$. The flat state trivially has $\mathbf{z} = 0$, as all $\rho = 0$; we want to find a continuous path $\rho_i(t)$ in folding angle space, parameterized by some variable t, beginning at the flat state and maintaining $\mathbf{z} = 0$.

In the spirit of perurbation theory we compute successive derivatives of $\mathbf{z}$ with respect to t, in order to set them to zero at $t = 0$:

$$\begin{aligned} \dot{\mathbf{z}}|_{t=0} &= 0 \\ \ddot{\mathbf{z}}|_{t=0} &= 0 \\ \dddot{\mathbf{z}}|_{t=0} &= 0 \\ &\vdots \end{aligned} \tag{8}$$

Henceforth we omit the designation $|_{t=0}$ as all derivatives are evaluated at $t = 0$.

Since at each order, four new variables enter (higher t derivatives of each $\mathbf{C}_i \equiv \cos(\rho_i)$), and four new quantities must be set to zero (the four components of the appropriate t derivative of $\mathbf{z}$), we might expect that there is exactly one solution for $\{\dot{\mathbf{C}}_i, \ddot{\mathbf{C}}_i, ...\}$ and our task is simply to find it. However, that solution is the trivial $\mathbf{C}_i(t) = 0$ for all t. In order to have a non-trivial solution, we must make a matrix corresponding to the highest derivatives singular: see below. But since these matrices appears in the equation at every order, solving each additional order requires the imposition of one more constraint.

Zeroth loop equation

The first equation is trivially satisfied for any path $\rho_i(t)$; to see this, note that

$$\dot{\mathbf{z}} = \frac{\partial \mathbf{z}}{\partial \mathbf{C}_i}\dot{\mathbf{C}}_i \tag{9}$$

where repeated indices are summed over.

Here it is worthwhile to note the two different 4-dimensional vector spaces with which we are working. $\mathbf{C}_i$ has $i = 1, 2, 3, 4$ running over the internal edges of the quad pattern shown above, while the four components of $\mathbf{z}$ are associated with the four vertices. Thus $\frac{\partial \mathbf{z}_a}{\partial \mathbf{C}_i}$ is a matrix operator from edge space to corner space, and higher derivatives are higher order tensor operators. Of course, the a, i component of this operator will be zero if edge i does not join vertex a.

Now from Eqn. 1, $\frac{\partial \mathbf{z}_a}{\partial \mathbf{C}_i}$ turns out to be zero at $\mathrm{C}_\alpha = \mathrm{C}_\beta = -1$ if the sum of the four in-plane angles at vertex a is 2π, as in the case we are considering. So any set of $\dot{\mathbf{C}}_i$s will satisfy this condition and we do not have any non-trivial constraint.

First loop equation

The first non-trivial condition is at second order (repeated indices summed over),

$$\ddot{\mathbf{z}} = \frac{\partial \mathbf{z}}{\partial \mathbf{C}_i}\ddot{\mathbf{C}}_i + \frac{\partial^2 \mathbf{z}}{\partial \mathbf{C}_i \partial \mathbf{C}_j}\dot{\mathbf{C}}_i\dot{\mathbf{C}}_j = \frac{\partial^2 \mathbf{z}}{\partial \mathbf{C}_i \partial \mathbf{C}_j}\dot{\mathbf{C}}_i\dot{\mathbf{C}}_j \tag{10}$$

Working out $\frac{\partial^2 \mathbf{z}}{\partial \mathbf{C}_i \partial \mathbf{C}_j}$, we find that $\ddot{\mathbf{z}}_a$ will be zero if $\dot{\mathbf{C}}_{a+1} = R_a \dot{\mathbf{C}}_a$ (where again R_a is the linear transfer coefficient between creases i and $i+1$). In other words, the equation above will be satisfied together if $\dot{\mathbf{C}}$ is a zero eigenvector of the matrix,

$$\begin{bmatrix} R_1 & -1 & 0 & 0 \\ 0 & R_2 & -1 & 0 \\ 0 & 0 & R_3 & -1 \\ -1 & 0 & 0 & R_4 \end{bmatrix}. \tag{11}$$

The first loop equation is thus obtained by imposing that the above matrix is singular (so that it has a non-trivial zero eigenvector):

$$\boxed{R_1 R_2 R_3 R_4 = 1} \tag{12}$$

where the explicit form of R is given in equation (4). If the first loop equation is satisfied, the solution $\dot{\mathbf{C}}$ that satisfies equation (10) obeys

$$\dot{\mathbf{C}}_{i+1} = R_i \dot{\mathbf{C}}_i, \tag{13}$$

For later use, we take $\dot{\mathbf{C}}$ to be normalized to have magnitude 1.

Second loop equation

Now, the third order derivative is

$$\dddot{\mathbf{z}} = \frac{\partial \mathbf{z}}{\partial \mathbf{C}_i} \dddot{\mathbf{C}}_i + 3 \frac{\partial^2 \mathbf{z}}{\partial \mathbf{C}_i \partial \mathbf{C}_j} \ddot{\mathbf{C}}_i \dot{\mathbf{C}}_j + \frac{\partial^3 \mathbf{z}}{\partial \mathbf{C}_i \partial \mathbf{C}_j \partial \mathbf{C}_k} \dot{\mathbf{C}}_i \dot{\mathbf{C}}_j \dot{\mathbf{C}}_k \tag{14}$$

$$= 3 \frac{\partial^2 \mathbf{z}}{\partial \mathbf{C}_i \partial \mathbf{C}_j} \ddot{\mathbf{C}}_i \dot{\mathbf{C}}_j + \frac{\partial^3 \mathbf{z}}{\partial \mathbf{C}_i \partial \mathbf{C}_j \partial \mathbf{C}_k} \dot{\mathbf{C}}_i \dot{\mathbf{C}}_j \dot{\mathbf{C}}_k \tag{15}$$

$$\equiv 3 M \ddot{\mathbf{C}} + \mathbf{v_{111}}. \tag{16}$$

Here we have made the definition $\mathbf{v_{111}} = \frac{\partial^3 \mathbf{z}}{\partial \mathbf{C}_i \partial \mathbf{C}_j \partial \mathbf{C}_k} \dot{\mathbf{C}}_i \dot{\mathbf{C}}_j \dot{\mathbf{C}}_k$, intended as a special case of the general definition

$$\mathbf{v_{p_1 p_2 \dots p_n}} = \frac{\partial^n \mathbf{z}}{\partial \mathbf{C}_{i_1} \partial \mathbf{C}_{i_2} \dots \partial \mathbf{C}_{i_n}} \frac{d^{p_1} \mathbf{C}_{i_1}}{dt^{p_1}} \frac{d^{p_2} \mathbf{C}_{i_2}}{dt^{p_2}} \dots \frac{d^{p_n} \mathbf{C}_{i_n}}{dt^{p_n}}. \tag{17}$$

We have also defined the matrix $M = \frac{\partial^2 \mathbf{z}}{\partial \mathbf{C}_i \partial \mathbf{C}_j} \dot{\mathbf{C}}_j$. Note that M has one vertex index (from $\mathbf{z}$) and one edge index i (from $\mathbf{C}_i$). Since $\dot{\mathbf{C}}$ is known, M and $\mathbf{v_{111}}$ are explicitly known: they can be written as

$$M = \begin{bmatrix} D_1 \dot{\mathbf{C}}_2 & -D_1 \dot{\mathbf{C}}_1 & 0 & 0 \\ 0 & D_2 \dot{\mathbf{C}}_3 & -D_2 \dot{\mathbf{C}}_2 & 0 \\ 0 & 0 & D_3 \dot{\mathbf{C}}_4 & -D_3 \dot{\mathbf{C}}_3 \\ -D_4 \dot{\mathbf{C}}_4 & 0 & 0 & D_4 \dot{\mathbf{C}}_1 \end{bmatrix}, \quad \text{and} \quad \mathbf{v_{111}} = -3 \begin{bmatrix} D_1 \dot{\mathbf{C}}_1 \dot{\mathbf{C}}_2 K_1 \\ D_2 \dot{\mathbf{C}}_2 \dot{\mathbf{C}}_3 K_2 \\ D_3 \dot{\mathbf{C}}_3 \dot{\mathbf{C}}_4 K_3 \\ D_4 \dot{\mathbf{C}}_4 \dot{\mathbf{C}}_1 K_4 \end{bmatrix} \tag{18}$$

with D, E, F and K define at a given vertex by

$$\boxed{K_i = \frac{\dot{\mathbf{C}}_i E + \dot{\mathbf{C}}_{i+1} F}{D},} \tag{19}$$

$$\text{with } D = \frac{C}{R} + \frac{B}{2}, \quad E = 4 s_{\alpha\beta}^3 s_{\beta\gamma} (c_{\alpha\beta} c_{\beta\gamma} - s_{\alpha\beta} s_{\beta\gamma}) s_{\delta\alpha}^2, \quad F = 4 s_{\alpha\beta}^3 s_{\beta\gamma}^2 s_{\delta\alpha} (c_{\alpha\beta} c_{\delta\alpha} - s_{\alpha\beta} s_{\delta\alpha}). \tag{20}$$

We know from equation (10) that M is singular. Hence, if we want $\dddot{\mathbf{z}}|_{t=0} = 0$, we need to ensure that $\mathbf{v_{111}}$ is in the range of M. The condition for this to lie in the range of M is that

$$\boxed{K_1 + K_2 + K_3 + K_4 = 0}. \tag{21}$$

This can be seen in various ways, including by making the ansatz

$$\ddot{\mathbf{C}} = \begin{bmatrix} \dot{\mathbf{C}}_1(\dot{\mathbf{C}}_2^2 K_1 + \dot{\mathbf{C}}_3^2(K_1 + K_2) + \dot{\mathbf{C}}_4^2(K_1 + K_2 + K_3)) \\ \dot{\mathbf{C}}_2(\dot{\mathbf{C}}_3^2 K_2 + \dot{\mathbf{C}}_4^2(K_2 + K_3) + \dot{\mathbf{C}}_1^2(K_2 + K_3 + K_4)) \\ \dot{\mathbf{C}}_3(\dot{\mathbf{C}}_4^2 K_3 + \dot{\mathbf{C}}_1^2(K_3 + K_4) + \dot{\mathbf{C}}_2^2(K_3 + K_4 + K_1)) \\ \dot{\mathbf{C}}_4(\dot{\mathbf{C}}_1^2 K_4 + \dot{\mathbf{C}}_2^2(K_4 + K_1) + \dot{\mathbf{C}}_3^2(K_4 + K_1 + K_2)) \end{bmatrix}. \tag{22}$$

We then have

$$3M\ddot{\mathbf{C}} = 3\begin{bmatrix} D_1\dot{\mathbf{C}}_1\dot{\mathbf{C}}_2((\dot{\mathbf{C}}_2^2 + \dot{\mathbf{C}}_3^2 + \dot{\mathbf{C}}_4^2)K_1 - \dot{\mathbf{C}}_1^2(K_2 + K_3 + K_4)) \\ D_2\dot{\mathbf{C}}_2\dot{\mathbf{C}}_3((\dot{\mathbf{C}}_3^2 + \dot{\mathbf{C}}_4^2 + \dot{\mathbf{C}}_1^2)K_2 - \dot{\mathbf{C}}_2^2(K_3 + K_4 + K_1)) \\ D_3\dot{\mathbf{C}}_3\dot{\mathbf{C}}_4((\dot{\mathbf{C}}_4^2 + \dot{\mathbf{C}}_1^2 + \dot{\mathbf{C}}_2^2)K_3 - \dot{\mathbf{C}}_3^2(K_4 + K_1 + K_2)) \\ D_4\dot{\mathbf{C}}_4\dot{\mathbf{C}}_1((\dot{\mathbf{C}}_1^2 + \dot{\mathbf{C}}_2^2 + \dot{\mathbf{C}}_3^2)K_4 - \dot{\mathbf{C}}_4^2(K_1 + K_2 + K_3)) \end{bmatrix}, \tag{23}$$

which is equal to $\mathbf{v_{111}}$ iff $K_1 + K_2 + K_3 + K_4 = 0$.

Third loop equation

The fourth order derivative is

$$\ddddot{\mathbf{z}} = \frac{\partial \mathbf{z}}{\partial \mathbf{C}_i}\dddot{\mathbf{C}}_i + 4\frac{\partial^2 \mathbf{z}}{\partial \mathbf{C}_i \partial \mathbf{C}_j}\ddot{\mathbf{C}}_i\dot{\mathbf{C}}_j + 3\frac{\partial^2 \mathbf{z}}{\partial \mathbf{C}_i \partial \mathbf{C}_j}\ddot{\mathbf{C}}_i\ddot{\mathbf{C}}_j + 6\frac{\partial^3 \mathbf{z}}{\partial \mathbf{C}_i \partial \mathbf{C}_j \partial \mathbf{C}_k}\ddot{\mathbf{C}}_i\dot{\mathbf{C}}_j\dot{\mathbf{C}}_k + \frac{\partial^4 \mathbf{z}}{\partial \mathbf{C}_i \partial \mathbf{C}_j \partial \mathbf{C}_k \partial \mathbf{C}_l}\dot{\mathbf{C}}_i\dot{\mathbf{C}}_j\dot{\mathbf{C}}_k\dot{\mathbf{C}}_l \tag{24}$$

$$= 4M\dddot{\mathbf{C}} + 3\mathbf{v_{22}} + 6\mathbf{v_{211}} + \mathbf{v_{1111}} = 0. \tag{25}$$

Once again, we can evaluate the vector terms explicitly:

$$3\mathbf{v_{22}} = 3\begin{bmatrix} C_1\ddot{\mathbf{C}}_1^2 + A_1\ddot{\mathbf{C}}_2^2 + B_1\ddot{\mathbf{C}}_1\ddot{\mathbf{C}}_2 \\ C_2\ddot{\mathbf{C}}_2^2 + A_2\ddot{\mathbf{C}}_3^2 + B_2\ddot{\mathbf{C}}_2\ddot{\mathbf{C}}_3 \\ C_3\ddot{\mathbf{C}}_3^2 + A_3\ddot{\mathbf{C}}_4^2 + B_3\ddot{\mathbf{C}}_3\ddot{\mathbf{C}}_4 \\ C_4\ddot{\mathbf{C}}_4^2 + A_4\ddot{\mathbf{C}}_1^2 + B_4\ddot{\mathbf{C}}_4\ddot{\mathbf{C}}_1 \end{bmatrix}, 6\mathbf{v_{211}} = -6\begin{bmatrix} E_1(2\ddot{\mathbf{C}}_1\dot{\mathbf{C}}_1\dot{\mathbf{C}}_2 + \ddot{\mathbf{C}}_2\dot{\mathbf{C}}_1^2) + F_1(2\ddot{\mathbf{C}}_2\dot{\mathbf{C}}_1\dot{\mathbf{C}}_2 + \ddot{\mathbf{C}}_1\dot{\mathbf{C}}_2^2) \\ E_2(2\ddot{\mathbf{C}}_2\dot{\mathbf{C}}_2\dot{\mathbf{C}}_3 + \ddot{\mathbf{C}}_3\dot{\mathbf{C}}_2^2) + F_2(2\ddot{\mathbf{C}}_3\dot{\mathbf{C}}_2\dot{\mathbf{C}}_3 + \ddot{\mathbf{C}}_2\dot{\mathbf{C}}_3^2) \\ E_3(2\ddot{\mathbf{C}}_3\dot{\mathbf{C}}_3\dot{\mathbf{C}}_4 + \ddot{\mathbf{C}}_4\dot{\mathbf{C}}_3^2) + F_3(2\ddot{\mathbf{C}}_4\dot{\mathbf{C}}_3\dot{\mathbf{C}}_4 + \ddot{\mathbf{C}}_3\dot{\mathbf{C}}_4^2) \\ E_4(2\ddot{\mathbf{C}}_4\dot{\mathbf{C}}_4\dot{\mathbf{C}}_1 + \ddot{\mathbf{C}}_1\dot{\mathbf{C}}_4^2) + F_4(2\ddot{\mathbf{C}}_1\dot{\mathbf{C}}_4\dot{\mathbf{C}}_1 + \ddot{\mathbf{C}}_4\dot{\mathbf{C}}_1^2) \end{bmatrix}, \mathbf{v_{1111}} = 6\begin{bmatrix} G_1\dot{\mathbf{C}}_1^2\dot{\mathbf{C}}_2^2 \\ G_2\dot{\mathbf{C}}_2^2\dot{\mathbf{C}}_3^2 \\ G_3\dot{\mathbf{C}}_3^2\dot{\mathbf{C}}_4^2 \\ G_4\dot{\mathbf{C}}_4^2\dot{\mathbf{C}}_1^2 \end{bmatrix} \tag{26}$$

where $G = -4s_{\alpha\beta}^4 s_{\beta\gamma}^2 s_{\delta\alpha}^2$. Let us define L_i by setting,

$$3\mathbf{v_{22}} + 6\mathbf{v_{211}} + \mathbf{v_{1111}} \equiv \begin{bmatrix} \dot{\mathbf{C}}_1\dot{\mathbf{C}}_2 D_1 L_1 \\ \dot{\mathbf{C}}_2\dot{\mathbf{C}}_3 D_2 L_2 \\ \dot{\mathbf{C}}_3\dot{\mathbf{C}}_4 D_3 L_3 \\ \dot{\mathbf{C}}_4\dot{\mathbf{C}}_1 D_4 L_4 \end{bmatrix} \tag{27}$$

By comparison with equations (19) and (21), we see that there will be a solution if we can write with

$$\boxed{L_1 + L_2 + L_3 + L_4 = 0}. \tag{28}$$

We can work out the explicit form of L_i from the above equations,

$$D_i L_i = 3(\frac{C_i}{R_i}\Gamma_i^2 + A_i R_i \Gamma_{i+1}^2 + B_i\Gamma_i\Gamma_{i+1}) - 6\left(E_i(2\dot{\mathbf{C}}_i\Gamma_i + \Gamma_{i+1}\dot{\mathbf{C}}_i) + F_i(2\dot{\mathbf{C}}_{i+1}\Gamma_{i+1} + \dot{\mathbf{C}}_{i+1}\Gamma_i)\right) + 6G_i\dot{\mathbf{C}}_i\dot{\mathbf{C}}_{i+1}, \tag{29}$$

$$\text{with } \Gamma_i = \dot{\mathbf{C}}_{i+1}^2 K_i + \dot{\mathbf{C}}_{i+2}^2(K_i + K_{i+1}) + \dot{\mathbf{C}}_{i+3}^2(K_i + K_{i+1} + K_{i+2}). \tag{30}$$

Note that here and hereafter, subscript indices $i+1$, $i+2$ and $i+3$ must be understood as modulo 4. Since $\Gamma_{i+1} = \Gamma_i - K_i$, we can write

$$D_i L_i = 3(\frac{C_i}{R_i}\Gamma_i^2 + A_i R_i(\Gamma_i - K_i)^2 + B_i\Gamma_i(\Gamma_i - K_i)) - 6(E_i(2\dot{\mathbf{C}}_i\Gamma_i + (\Gamma_i - K_i)\dot{\mathbf{C}}_i) + F_i(2\dot{\mathbf{C}}_{i+1}(\Gamma_i - K_i) + \dot{\mathbf{C}}_{i+1}\Gamma_i)) + 6G_i\dot{\mathbf{C}}_i\dot{\mathbf{C}}_{i+1}.$$

Using the definitions of R, K and D this further simplifies

$$\boxed{L_i = -12K_i\Gamma_i + \frac{3A_iR_iK_i^2}{D_i} + 6K_i^2 + 6\frac{F_iK_i}{D_i}\dot{\mathbf{C}}_{i+1} + 6\frac{G_i}{D_i}\dot{\mathbf{C}}_i\dot{\mathbf{C}}_{i+1}}. \tag{31}$$

Generating arbitrary loop equations

The nth order derivative of $\mathbf{z}$ has two types of term. The first is the single term $nM\frac{d^{n-1}\mathbf{C}}{dt^{n-1}}$. The second is terms of the form $\mathbf{v_{p_1(n-p_1)}}$, $\mathbf{v_{p_1p_2(n-p_1-p_2)}}$ and $\mathbf{v_{p_1p_2p_3(n-p_1-p_2-p_3)}}$, with numerical factors that can be straightforwardly determined by combinatorics. Importantly, terms of this form can have at most 4 indices, because the equation for $\mathbf{z}$ is a fourth order polynomial in the $\mathbf{C}_i$s. Furthermore, the highest t derivative of a $\mathbf{C}_i$ that appears in these terms is $n-2$, and the first $n-2$ derivatives of $\mathbf{C}$ were calculated using the equations arising from the first $n-1$ derivatives of $\mathbf{z}$. Thus the equation arising from setting the nth derivative of $\mathbf{z}$ to zero is

$$nM\frac{d^{n-1}\mathbf{C}}{dt^{n-1}} + \mathbf{V^n} = 0, \tag{32}$$

where $\mathbf{V^n}$ is the sum of known vectors. As in the derivation of the second and third loop equations, we then define $\mathbf{L^n}$ by

$$V_i^n = \dot{\mathbf{C}}_i\dot{\mathbf{C}}_{i+1}D_iL_i^n, \tag{33}$$

and the $n-1$th loop equation is $L_1^n + L_2^n + L_3^n + L_4^n = 0$. Finally,

$$\frac{d^{n-1}\mathbf{C}_i}{dt^{n-1}} = \dot{\mathbf{C}}_i(\dot{\mathbf{C}}_{i+1}^2L_i^n + \dot{\mathbf{C}}_{i+2}^2(L_i^n + L_{i+1}^n) + \dot{\mathbf{C}}_{i+3}^2(L_i^n + L_{i+1}^n + L_{i+2}^n)). \tag{34}$$

MATLAB code to generate such equations is provided as supplementary material.

Supplementary Note 2 - Flat foldability, rigid foldability and the loop equations

Flat-foldability

A flat foldable pattern is an origami pattern that can be folded completely flat down to a plane. Kawasaki-Justin showed that a necessary condition for a single 4-vertex to be flat-foldable is that opposing in-plane angles at the vertex sum to π. (Bern and Hayes[4] showed that no simple global criterion exists to check if a pattern is globally flat foldable, i.e., avoids global collisions while folding flat.)

Despite the name, flat foldability of vertices does not imply any foldability of a crease pattern made of such vertices. In fact, such a crease pattern may not be foldable even to first order! If one would like to reach the flat-folded state of a generic crease pattern with flat foldable 4-vertices, the system would have to be "snapped" through states with large face bending, requiring energy input.

Flat-foldable vertices are more restricted than general vertices in the number of design parameters available and the folded geometries they can acquire. Flat-foldable vertices have only two independent design parameters (i.e., in-plane angles) at each vertex since opposite angles need to sum to π; in contrast, general vertices studied in this paper have three independent in-plane angles. Further, the folded geometries that a flat-foldable vertex can acquire is limited; the four folding angles $\rho_1, \rho_2, \rho_3, \rho_4$ at a vertex are constrained such that $|\rho_1| = |\rho_3|, |\rho_2| = |\rho_4|$. That is, opposite creases are forced to fold to the same extent. In contrast, general vertices can achieve arbitrary folding angles $\rho_1, \rho_2, \rho_3, \rho_4$, allowing more kinds of local geometry.

Tachi's loop equation for flat-foldable quads

Tachi identified a single loop equation as a necessary and sufficient condition for quads made of flat foldable vertices to also be rigidly foldable with no face bending at all to any order[5]. Tachi later derived a 1-st order loop equation for general non-flat foldable quads[6]. In contrast, we studied general quads with possibly non-flat foldable vertices and found that general patterns are faced with a hierarchy of loop equations. Solving these in sequence produces a more and more foldable pattern over orders of magnitude.

Our results for tunable foldibility of general crease patterns with arbitrary Mountain-Valley choice relate to Tachi's work on rigid foldability of crease patterns with 'flat-foldable' vertices. For quads with flat foldable vertices, the first of our loop equations, $\Pi R = 1$ is the only independent equation. Solving it immediately solves all other equations. Thus, for quads with flat foldable vertices, first order foldability implies rigid foldability. This is consistent with Tachi's result that there is only one loop equation for quads with flat foldable vertices. We have also numerically verified that quads with flat foldable vertices satisfying Tachi's loop equation automatically satisfy all of our loop equations.

The amount of rigid foldable patterns

To be rigidly foldable with no face bending, the in-plane angles of a quad must solve our infinite hierarchy of loop equations. Since a quad has only 11 independent design parameteres (i.e., in-plane angles θ), no rigidly foldable quads can be expected to exist unless the loop equations are somehow not independent.

We verified that the first **five** of our loop equations are indeed independent; for example, as quads solve the fourth loop equation $\Sigma M = 0$ more and more precisely, the residue of the fifth equation ΣN stays finite. Thus, the space of rigidly foldable quad patterns is at most $11 - 5 = 6$ parameter. Combined with Tachi's results showing a 6 parameter space of rigidly foldable quads, this indicates that higher loop equations, beyond the 5th loop equation, are not actually independent. (To see this, note that 'flat foldable' quads studied by Tachi[5] have 7 independent design parameters. On imposing Tachi's single loop equation needed for rigid foldability, one obtains a 6 parameter family of rigidly foldable quads.)

To summarize, our work shows that space of rigidly foldable quads is no more than a 6 parameter family and Tachi's earlier work[5] already demonstrated the existence of a 6 parameter rigidly foldable family.

Other rigidly foldable patterns:

In principle, there could exist other 6 parameter (or lower) families of rigidly foldable quads that are not flat foldable and hence not in Tachi's family. Indeed, non-flat foldable but rigidly foldable quads can easily exist due to symmetry. For example, the trapezoid-like crease pattern (Supplementary Fig. 1c) shows a member of a 5-parameter family of quads that is rigidly foldable due to symmetry. The reflection symmetry in these trapezoids means that the transfer functions cancel to all orders in pairs of adjacent vertices. Besides such solutions due to symmetry, we were not able to find any rigidly foldable non-flat foldable quads by sampling random quads and solving loop equations by gradient descent. While such numeric searches cannot mathematically rule out the possibility of rigidly foldable quads (besides those described by Tachi) that are solutions of all our loop equations, any such rigidly foldable quads cannot constitute more than a 6-parameter family.

Supplementary Methods

Manufacturing origami prototypes

We made origami prototypes by cutting 120 lb cardstock using a laser cutter. Patterns were roughly of size 10 cm x 10 cm. Creases were created using a perforation pattern of 0.6 mm cuts with 0.7 mm gaps. These patterns were folded by hand according to their respective designed MV configurations. Prototypes in Fig. 1b and 1e were recolored using Adobe Photoshop to ensure distinct colors in Fig. 1.

Folding using constraint matrices

We simulate the folding of rigid origami using the constraint matrices introduced by Tachi[7]. For each vertex with edges e_i, in-plane angles θ_{ij} and creases folded to a general set of fold angles ρ_i. Define $A_{ij} = R(\theta_{ij}, n_{ij})$, the $SO(3)$ rotation matrix by θ_{ij} degrees about the normal n_{ij} to the face between edge i and j (i.e., $n_{ij} = e_i \times e_j$ where e_i and e_j are unit vectors along the edges). Let $B_i = R(\rho_i, e_i)$ be the rotation matrix about edge i by an angle ρ_i. If the vertex is folded without any tearing, gaps or bending of faces between edges, the product of these rotation matrices should bring us back to the origin; $F(\{\rho_i\}) = A_{12}B_2A_{23}B_3A_{34}B_4A_{41}B_1 = \mathbf{I}$. If a small change in fold angles $\delta\rho$ does not violate these constraints, it must satisfy, $\delta F = \sum_i \frac{\partial F}{\partial \rho_i}\delta\rho_i = 0$. Note that F is an $SO(3)$ matrix and hence δF is a skew-symmetric $so(3)$ matrix. Hence we can define $C_{ai} = \frac{\epsilon_{abc}\delta F_{bc}}{\delta\rho_i}$ which allows us to re-write the constraint equation as $C(\rho)\delta\rho = 0$.

Thus, the allowed motions $\delta\rho$ at any given folded state ρ are given by the zero modes of $C(\rho)$. Hence we solve the differential equation $\dot{\rho} = (\mathbf{I} - C^{+}C)\tau_{\text{app}}$ where τ_{app} is set of folding torques applied to creases and C^{+} is the pseudo-inverse of C. Here $\mathbf{I} - C^{+}C$ projects the change of angles caused by τ_{app} to the null space of C. We solve the above equation using MATLAB's ODE solver.

Sampling random simple / augmented quads

A simple quad is uniquely defined by specifying the coordinates of 12 points on a plane. Provided the points are connected through non-intersecting lines, the resulting graph will describe a simple quad (Supplementary Fig. 2d). The same set of points also uniquely define the augmented quad, with the convention that the center diagonal is drawn from the bottom left to the top right (Supplementary Fig. 2d).

Each simple quad is sampled by first setting the 12 points to define a perfect square lattice of unit length 1. Then, both coordinates of each point are altered randomly by adding a number drawn out of a uniform distribution on $[-0.5, 0.5]$. The resulting quad is guaranteed to have a convex quadrilateral at its center, so the corresponding augmented quad can be constructed using the same coordinate set.

A large mesh is sampled similarly, using a larger square lattice as the initial state, e.g., a 2×2 quads mesh starts with a 4×4 square lattice. Lattice points are then displaced using a random uniform distribution. Finally, the center face of each quad is augmented with a diagonal crease.

Detection of folding modes

For each augmented quad (or mesh) sampled by the method described above, we obtain a list of all zero energy modes by applying random normalized torques to the flat state, and folding it using the constraint matrix method. As the simple quad is made of 12 creases, the torque on the quad is a 12-component vector, one value for every crease. After sampling a number of random torques and recording the resulting folding modes, we sample an additional number of torques and record more unique modes. This step is repeated as long as new unique zero modes are found. By this procedure (nearly) all zero modes are detected, barring those corresponding to small surface areas on the 12-sphere. For larger quad meshes, we carry out the same procedure but with torques vectors of length corresponding to the number of creases in the pattern.

Foldability along a folding mode

Sampled augmented quads were analyzed to find their zero modes, in particular those that are compatible with the corresponding simple quads. However, the existence of a zero mode informs us little about how it behaves when folding the quad. In particular, it is known there exist quads that are bistable (having stable configurations divided by modes that are not precise zero modes). In our setting, bistable modes would have face folding grow up to some angle and then overturn. Such behavior would seem to indicate that the more folded configuration costs less energy.

We study a few of the sampled quads and analyze all of their legal modes (those compatible with the corresponding simple quad). To improve numerical stability, simulation is started in the folded state where the maximum crease angle is 1 Rad. The pattern is then folded backwards toward the flat states at intervals of 0.05 Rad, and forward towards π using the same interval. We note that patterns are folded until the maximum crease angle reaches 3.1 Rad. Folding very close to angle π is avoided, as this angle value indicates a collision of faces, after which the system leaves the domain of validity of our analysis.

Supplementary References

[1] Dudte, L. H., Vouga, E., Tachi, T. & Mahadevan, L. Programming curvature using origami tessellations. *Nat Mater* (2016).

[2] Silverberg, J. L. *et al.* Origami structures with a critical transition to bistability arising from hidden degrees of freedom. *Nat Mater* **14**, 389–393 (2015).

[3] Waitukaitis, S., Menaut, R., Chen, B. G.-g. & van Hecke, M. Origami multistability: From single vertices to metasheets. *Physical review letters* **114**, 055503 (2015).

[4] Bern, M. & Hayes, B. The complexity of flat origami. *Proceedings of the 7th Annual ACM-SIAM Symposium on Discrete Algorithms* 175–183 (1996).

[5] Tachi, T. Generalization of rigid foldable quadrilateral mesh origami. *Proceedings of the International Association for Shell and Spatial Structures (IASS) Symposium* (2009).

[6] Tachi, T. Design of infinitesimally and finitely flexible origami based on reciprocal figures. *Journal for Geometry and Graphics* **16**, 223–234 (2012).

[7] Tachi, T. Simulation of rigid origami. *Origami* **4**, 175–187 (2009).
